# Investigating the Implications of *CFTR* Exon Skipping Using a *Cftr* Exon 9 Deleted Mouse Model

**DOI:** 10.3389/fphar.2022.868863

**Published:** 2022-03-22

**Authors:** Kelly M. Martinovich, Anthony Kicic, Stephen M. Stick, Russell D. Johnsen, Sue Fletcher, Steve D. Wilton

**Affiliations:** ^1^ School of Medicine, The University of Western Australia, Perth, WA, Australia; ^2^ Telethon Kids Institute, Wal-yan Respiratory Research Centre, Perth, WA, Australia; ^3^ Centre for Molecular Medicine and Innovative Therapeutics, Health Futures Institute, Murdoch University, Murdoch, WA, Australia; ^4^ Centre for Cell Therapy and Regenerative Medicine, School of Medicine and Pharmacology, The University of Western Australia, Perth, WA, Australia; ^5^ Department of Respiratory and Sleep Medicine, Perth Childrens Hospital, Nedlands, WA, Australia; ^6^ School of Population Health, Curtin University, Bentley, WA, Australia; ^7^ Perron Institute for Neurological and Translational Sciences, Centre for Neuromuscular and Neurological Disorders, The University of Western Australia, Nedlands, WA, Australia; ^8^ PYC Therapeutics, Perth, WA, Australia

**Keywords:** mouse model, cystic fibrosis transmembrane conductance regulator, exon skipping therapy, transgenic mouse, exon deletion

## Abstract

**Introduction:** Severity and disease progression in people with Cystic Fibrosis (CF) is typically dependent on their genotype. One potential therapeutic strategy for people with specific mutations is exon skipping with antisense oligonucleotides (AO). *CFTR* exon 9 is an in-frame exon and hence the exclusion of this exon would excise only 31 amino acids but not alter the reading frame of the remaining mRNA. Splice mutations 1209 + 1 G > C and 1209 + 2 T > G were documented to cause *CFTR* exon 9 skipping and these variants were reported to manifest as a milder CF disease, therefore exon 9 skipping could be beneficial for people with class I mutations that affect exon 9 such as p.Trp401X. While the impact of exon 9 skipping on gene expression and cellular pathways can be studied in cells *in vitro*, trace amount of full-length normal or mutated material could confound the evaluation. To overcome this limitation, the impact of *CFTR* exon 9 skipping on disease phenotype and severity is more effectively evaluated in a small animal model. It was hypothesised that antisense oligonucleotide-mediated skipping this particular exon could result in a “mild mouse CF phenotype”.

**Methods:** *Cftr* exon 9 deleted mice were generated using homologous recombination. Survival of homozygous (*Cftr* ^*Δ9/Δ9*^) and heterozygous (*Cftr* ^*Δ9/+*^) mice was compared to that of other CF mouse models, and lung and intestinal organ histology examined for any pathologies. Primary airway epithelial cells (pAECs) were harvested from *Cftr* ^*Δ9/Δ9*^ mice and cultured at the Air Liquid Interface for CFTR functional assessment using Ussing Chamber analysis.

**Results:** A *Cftr* ^*Δ9/Δ9*^ mouse model presented with intestinal obstructions, and at time of weaning (21 days). *Cftr* ^*Δ9/Δ9*^ mice had a survival rate of 83% that dropped to 38% by day 50. Histological sections of the small intestine from *Cftr* ^*Δ9/Δ9*^ mice showed more goblet cells and mucus accumulation than samples from the *Cftr* ^*Δ9/+*^ littermates. Airway epithelial cell cultures established from *Cftr* ^*Δ9/Δ9*^ mice were not responsive to forskolin stimulation.

**Summary:** The effect of *Cftr* exon 9 deletion on Cftr function was assessed and it was determined that the encoded Cftr isoform did not result in a milder “mouse CF disease phenotype,” suggesting that *Cftr* exon 9 is not dispensable, although further investigation in human CF pAECs would be required to confirm this observation.

## Introduction

Severity and disease progression in people with Cystic Fibrosis (CF) is typically dependent on their genotype. One personalised therapeutic option is the use of exon skipping antisense oligonucleotides (AO) that are based on a specific genotype (Radpour et al., 2007; Shteinberg et al., 2017; Gaikwad et al., 2018). A review of the literature led to two observations that have suggested partial redundancy of *CFTR* exon 9: 1) splice mutations 1209 + 1 G > C and 1209 + 2 T > G were documented to cause *CFTR* exon 9 skipping and these variants were reported to manifest as a milder form of disease caused by mutations in the *CFTR* gene; congenital bilateral absence of vas deferens (CBAVD) (Li et al., 2012) and 2) *CFTR* exon 9 is in-frame and is reported to carry relatively few CF-causing mutations, when compared to other *CFTR* exons (Toronto 2011; Henrie et al., 2018; Molinski et al., 2018). *CFTR* exon 9 is an in-frame exon therefore the exclusion of this exon would not alter the reading frame of the remaining mRNA. This strategy could be beneficial for people with class I “null mutations” in exon 9 such as p.Trp401X that prevent synthesis of a functional protein. While the impact of exon 9 skipping on gene expression and cellular pathways can be studied in cells *in vitro*, trace amounts of full-length normal or mutated material could confound the testing. To overcome this limitation, the impact of *CFTR* exon 9 skipping on disease phenotype and severity should be more effectively evaluated in a small animal model.

Typically, mice homozygous for a *Cftr* mutation or disrupted *Cftr* gene display many features common to a young person with CF caused by two severe mutations (ie p.Phe508del), including failure to thrive, meconium ileus, alteration of mucus and serous glands, and obstruction of gland-like structures with thickened eosinophilic material (Lavelle et al., 2016). Genetically modified mouse models of CF disease were initially created by homologous recombination in embryonic stem cells through targeting a mutation to a specific site in the murine genome, each with varied outcomes (Clarke et al., 1994; Gosselin et al., 1998) (Davidson and Rolfe 2001). (Davidson and Rolfe 2001). Typically, CF-associated death in mouse models with specific *Cftr* mutations was from intestinal obstruction, usually before 40 days of age (Snouwaert et al., 1992). Some CF mouse models used gene insertions without loss of genomic sequence therefore, potentially allowing a small amount of normal *Cftr* mRNA to be expressed that may contribute to a reduced severity of disease (Davidson and Rolfe 2001; Scholte et al., 2004). There are ‘two residual function’ CF mouse models, *Cftr* ^tm/Hgu^ and *Cftr* ^tm/Bay^ that show 90 and 40% survival respectively, but also have 10 and 2% of wild type (WT) *Cftr* mRNA present that would contribute to their higher survival rates (O'Neal et al., 1993; Wilke et al., 2011). Furthermore, three mouse models with the p.Phe508del mutation exist, showing variable WT CFTR mRNA levels and survival rates (5–90% survival at weaning) (Colledge et al., 1995; Zeiher et al., 1995; Rozmahel et al., 1996; Davidson and Rolfe, 2001).

Some transgenic CF mouse models used a gene replacement strategy to disrupt the *Cftr* gene creating absolute nulls. These complete *Cftr* KO models have no WT mRNA detected, such as *Cftr* ^tm/Unc^; *Cftr* ^tm/Cam^; *Cftr* ^tm/Hsc^; *Cftr* ^tm3Bay^; *Cftr* ^tm3Uth^ and have also shown variable survival rates after weaning (5–40%) (Colledge et al., 1995). *Cftr* mouse models generated with various mutations that result in nonsense mediated decay led to an absence of the functional Cftr protein (Snouwaert et al., 1992; O'Neal et al., 1993; Ratcliff et al., 1993; Hasty et al., 1995; Rozmahel et al., 1996; Wilke et al., 2011). One of these CF KO mouse models was generated with a premature termination codon in *Cftr* exon 2 (*Cftr* ^m3/Bay^) and resulted in intestinal obstruction, which led to death in 60% of the mutant animals within 1 month after birth (Hasty et al., 1995).

The β-ENaC mouse model was the first to recapitulate the pathophysiology of the CF lung. This model over-expresses the β subunit of the ENaC with increased sodium ion absorption. Mice present with reduced airway surface liquid, reduced mucociliary clearance with airway mucus obstruction, goblet cell hyperplasia, chronic lung inflammation, and high mortality related to the lung disease (Zhou et al., 2011). Although these differences reinforce the notion that mouse models do not accurately recapitulate the human disease phenotype or progression, they can provide important mechanistic data.

We hypothesised that *CFTR* exon 9 could be partially redundant, meaning the encoded amino acids might not be essential for full CFTR function, therefore enabling an exon skipping AO strategy to be therapeutic for people with amenable mutations in that exon. Skipping of *CFTR* exon 9 would not disrupt the reading frame and if the resultant isoform retained some function, that could result in a “milder mouse CF phenotype” when compared to complete KO models. To investigate this possibility, a *Cftr* exon 9 deleted mouse model (*Cftr* ^*Δ9/Δ9*^) was generated using homologous recombination that has no residual WT mRNA. Survival rates were compared to other CF mouse models and lung and intestinal organ histology examined for any pathologies. Primary airway epithelial cells (pAECs) were also harvested from *Cftr* ^*Δ9/Δ9*^ mice and cultured at the air liquid interface (ALI) for CFTR functional assessment using Ussing Chamber analysis.

## Methods

### Generation of *Cftr* ^*Δ9/Δ9*^ Mouse Model

The *Cftr* ^*Δ9/Δ9*^ mouse line was generated by Ozgene Pty Ltd. (Bentley WA, Australia) and approved by Murdoch University Animal ethics committee (Protocol ID: R3109/19). The targeting construct was electroporated into a C57BL/6 embryonic stem cell line, *Bruce4* (Koentgen et al., 2016). Homologous recombinant embryonic stem cell clones were identified by quantitative PCR and injected into goGermline blastocysts (Ozgene) (Koentgen et al., 2016). Male chimeric mice were obtained and crossed to C57BL/6J females to establish heterozygous germline offspring on the C57BL/6 background. The germline mice were crossed to a ubiquitous Cre C57BL/6 mouse line to remove the loxP flanked sequence. *Cftr* ^*Δ9/Δ9*^ were generated through heterozygous breeding and pups were genotyped at birth. Since feed and housing conditions have been documented to influence the survival of CF mouse models (Snouwaert et al., 1992; Cottart et al., 2007), after weaning (21 day) *Cftr* ^*Δ9/Δ9*^ mice were fed exclusively with Peptamen® (Nestle Health Science) 1 kcal/ml peptide based liquid feed to prevent gastrointestinal occlusions (Snouwaert et al., 1992). All mice were housed on ALPHA-dri® bedding (Shepherd Specialty Papers). Mice were monitored as per routine husbandry guidelines for up to 150 days and if euthanasia was required before 150 days due to intestinal obstruction or malnutrition, tracheas were harvested for pAEC cultures and lungs, intestines, heart, kidneys and liver were fixed in 4% paraformaldehyde (Sigma-Aldrich). Euthanasia was done by inhaled anaesthetic followed by cervical dislocation. Mice were not routinely weighed.

### Histology

Organs such as the lungs, intestines, heart, kidneys and liver were fixed in 4% paraformaldehyde and were removed from fixative and placed in a standard histology cassette for processing. After overnight dehydration, tissue blocks were processed for paraffin embedding using a Leica tissue processor. After processing, specimens were placed into the embedding station wax bath. Sections (5 µm) were cut using the Leica RM2125 RTS The Essential Microtome (Leica Biosystems) and allowed to bake at 60°C for 30 min, after which they were stained with Hematoxylin and Eosin (H & E) (Sigma-Aldrich) or Alcian Blue (Sigma-Aldrich) using a Leica Autostainer and visualised using an Olympus BX53 microscope with DP72 camera and images captured using NIS-Elements software.

### Mouse Airway Epithelial Cell Collection

Tracheas were harvested and digested in 1.5 mg/ml pronase as previously described (Lam et al., 2011). Briefly, tracheas were harvested from mice and placed into 30 ml of Ham’s F12 medium 1% penicillin/streptomycin and 1% fungizone and transferred to a sterile laminar flow hood. Tracheas were then cut along the vertical axis to expose the lumen and transferred to a new 50 ml tube containing 10 ml of Hams’s F12 medium (GIBCO) with antibiotics and 1.5 mg/ml pronase and incubated on a roller at 4°C overnight. The following day, 10 ml of Ham’s F12 medium containing 20% FBS and antibiotics were added, and the tube inverted 12 times. Three tubes containing Ham’s F12 medium, 20% FBS and antibiotics were prepared, and tracheas transferred to the first tube and then inverted 12 times and repeated twice more before the remaining tissue was discarded. All solutions were then combined and centrifuged at 1,400 rpm for 10 min at 4°C. The cell pellet was resuspended in 1 ml of DNAse solution containing 10 mg/ml BSA, 10 mg/ml DNAse I (Roche) made up in Hams’s F12 medium containing antibiotics and incubated for 5 min on ice. Cells were then centrifuged at 1,400 rpm for 10 min at 4°C, resuspended in mouse tracheobronchial epithelial cell (MTEC) medium containing 10% FBS and plated into a plastic tissue culture dish before incubation at 37°C, 95% air, 5% CO_2_ for 5 h to negatively select fibroblasts (Lam et al., 2011). Afterwards, the supernatant was collected, and the dish was rinsed with 4 ml of MTEC medium containing 10% FBS, that was then pooled with the supernatant and centrifuged at 1,400 rpm for 10 min at 4°C. Cell pellets were then resuspended for appropriate primary cell culture seeding.

### Mouse Airway Epithelial Cell Culture and Functional CFTR Assessment

For monolayer epithelial cells cultures, harvested tracheal cells were resuspended in 4 ml MTEC basic media (Lam et al., 2011) and plated into 6 well plates, precoated with 50 μg/ml collagen I and incubated at 37°C, in 95% air, 5% CO_2_. Once confluent, cells were trypsinised using the ReagentPack™ Subculture Reagents (Lonza™) according to the manufacturer’s instructions. Cells were then centrifuged at 1,400 rpm for 10 min at 4°C before the pellet was resuspended in 1 ml of MTEC proliferation media (Lam et al., 2011) and a total cell count performed. Cells (300,000) were seeded into collagen I pre-coated apical compartments of a Corning® Costar® 12 mm Snapwell™ cell culture insert with 0.4 µm pore polycarbonate membrane (3,407 Sigma), as previously described (Martinovich et al., 2017). To complete the culture, 2 ml of MTEC proliferation medium was added to the basolateral compartment and cultures incubated at 37°C, 95% air, 5% CO_2_. Cultures were monitored and media replaced daily. Once cells were confluent, the apical compartment media was removed and not replaced, exposing the insert to air, creating an air liquid interface (ALI) and allowing the cells to fully differentiate. Once airlifted, the basolateral MTEC proliferation media was supplemented with 2% v/v Ultroser™ G serum substitute (15,950–017 Pall) and 0.1 µM Trans-retinoic acid and changed every second day. To monitor monolayer differentiation transepithelial electrical resistance (TEER) measurements were performed twice weekly with STX2 chopstick electrodes and Epithelial Voltohmmeter EVOM^2^ (World Precision Instruments). Ussing chamber (Physiologic Instruments Inc.) analysis was performed on day 28 post ALI, as previously described (Martinovich et al., 2017). Briefly, A chloride ion gradient was established by filling the basolateral compartment with Krebs Ringer bicarbonate solution as previously described (Thomas et al., 2000). Chambers were manipulated with Amiloride (50 µM Sigma-Aldrich), Forskolin (0.2–20 µM F6886 Sigma-Aldrich), CFTR-inhibitor 172 (30 µM 219,670 Sigma-Aldrich) and Ivacaftor (3 µM VX-770, Selleckchem). Inserts were pre-treated with either Lumacaftor (3 µM VX-809, Selleckchem) or DMSO (0.001%, Sigma-Aldrich) 24 h prior to Ussing Chamber analysis. Tracings were recorded using Acquire and Analyse 2.3 software (Physiologic Instruments Inc.) and raw values exported to excel. The short circuit current (Isc) data was then visualised using GraphPad Prism 8 software. For magnitude of change in Isc due to drug manipulations, the stable values immediately before and after the change were recorded and the differences plotted using GraphPad Prism 8 software.

## Results

### *Cftr* ^*Δ9/Δ9*^ Mice

**FIGURE 1:**
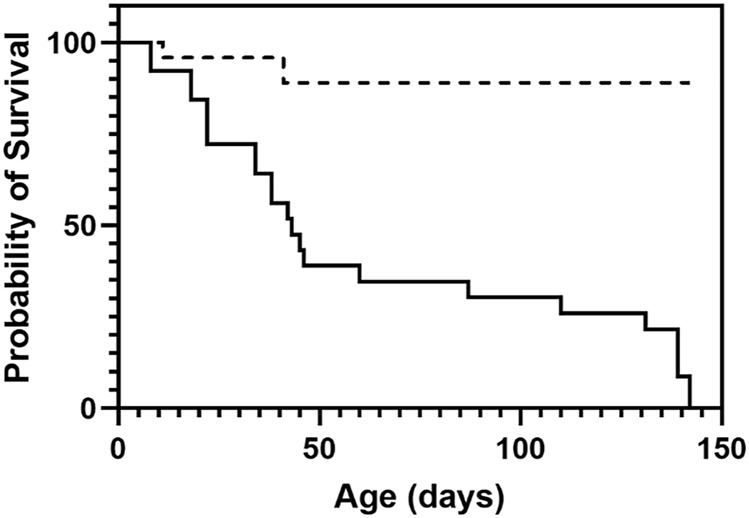
Survival plot of *Cftr* ^*Δ9/Δ9*^ mice. At time of weaning (21 days) *Cftr* ^*Δ9/Δ9*^ mice had a survival of 83% which dropped to 38% by day 50, with most mice succumbing to intestinal obstruction or malnutrition (solid line). Heterozygous mice did not show decreased survival with 89% survival at day 50 until day 150 when monitoring ended (dashed line). *n* = 24.

*Cftr* ^*Δ9/Δ9*^ pups were successfully generated from heterozygous breeding that resulted in 107 pups (12%) that were *Cftr* ^*Δ9/Δ9*^ *, 31% Cftr* ^*+/+*^ and 57% *Cftr* ^*Δ9/+*^ *.* Homozygous *Cftr* ^*Δ9/Δ9*^ pups were supplied with Peptamen food at approximately day 14 and weaned around day 21. At time of weaning (21 days) *Cftr* ^*Δ9/Δ9*^ mice had a survival rate of 83%. By day 50, more than half of the *Cftr* ^*Δ9/Δ9*^ mice had signs of intestinal obstruction or malnutrition and were humanely euthanised (Figure 1) compared to the 89% event free survival rate of the *Cftr* ^*Δ9/+*^ littermates (*n* = 24, 14 Male and 10 Female). Mice were not routinely weighed, however *Cftr* ^*Δ9/Δ9*^ mice were noted to have a “scruffier” appearance and “a general failure to thrive” by Ozgene staff members who were maintaining the colony.

**FIGURE 2:**
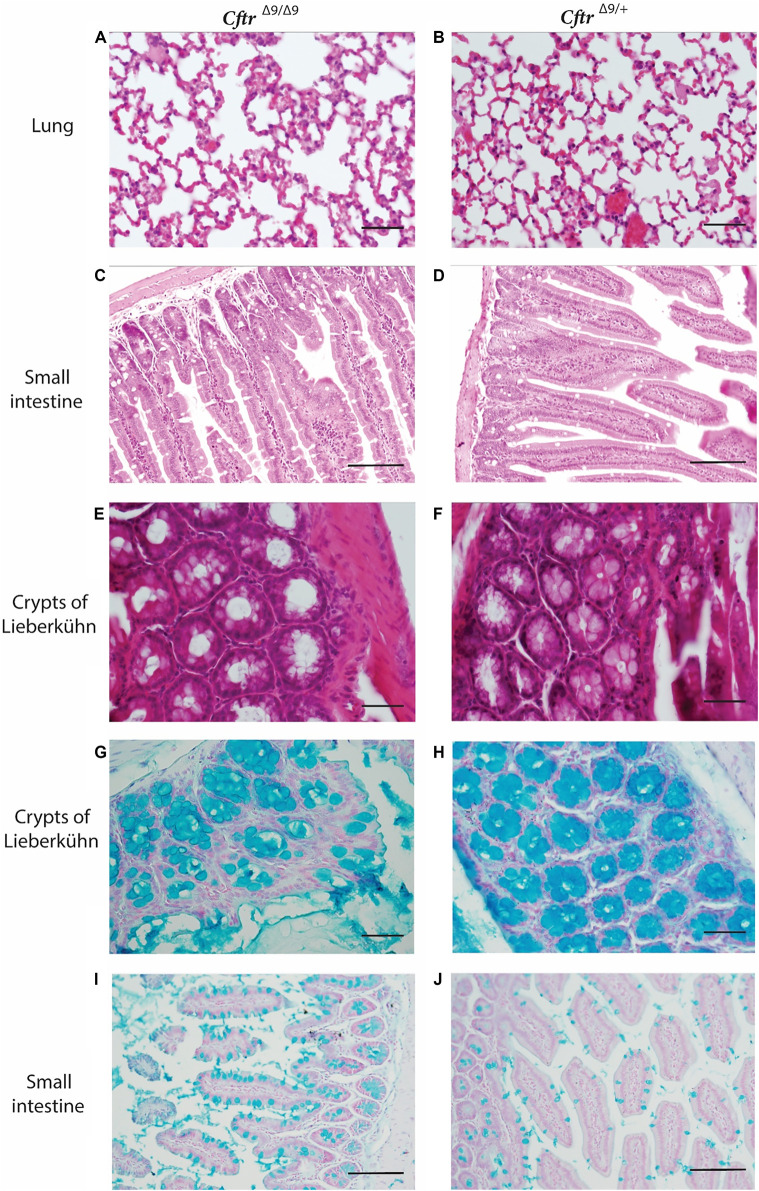
Hematoxylin and Eosin and Alcian Blue staining of representative lung and intestinal sections from *Cftr* ^*Δ9/Δ9*^ and *Cftr* ^*Δ9/+*^ (control) mice. **(A–F)** Hematoxylin and Eosin stained **(A)**. Lung section for *Cftr* ^*Δ9/Δ9*^ mouse. **(B)**. Lung section for *Cftr* ^*Δ9/+*^ mouse. **(C)**. Small intestinal section from *Cftr* ^*Δ9/Δ9*^ mouse. **(D)**. Intestinal section from *Cftr* ^*Δ9/+*^ mouse. **(E)**. Crypts of Lieberkühn section from *Cftr* ^*Δ9/Δ9*^ mouse. **(F)**. Lieberkühn section from *Cftr* ^*Δ9/+*^ *m*ouse. **(G–J)** Alcain blue stained **(G)**. a *Cftr* ^*Δ9/Δ9*^ mouse and **(H)**. *Cftr* ^*Δ9/+*^ mouse showing Crypts of Lieberkühn. Sections of small intestine from **(I)**. *Cftr* ^*Δ9/Δ9*^ mouse and **(J)**. *Cftr* ^*Δ9/+*^ mouse, showing goblet cells. *n* = 4. Images **(A,B)**, **(E–H)** were taken at ×40 (scale bar 20 micrometres) and **(C,D)**, **(I,J)** were taken at ×20 (scale bar 50 micrometres).

To identify any pathological changes in the lungs of the *Cftr* ^*Δ9/Δ9*^ mice, the lungs were excised and prepared for histology and H & E staining (*n* = 4/group, 2 Male and 2 Female/group). The age of the mice at time of collection varied, dependant on when the *Cftr* ^*Δ9/Δ9*^ animals met the humane endpoint and ranged from 21 to 150 days of age, with *Cftr* ^*Δ9/+*^ mice age matched where possible. Upon examination, no obvious lung fibrosis or mucus accumulation was evident in the homozygous *Cftr* ^*Δ9/Δ9*^ lungs or their *Cftr* ^*Δ9/+*^ littermates (Figures 2A,B). Macroscopic examination showed homozygous *Cftr* ^*Δ9/Δ9*^ mice had intestinal obstruction to some degree at the time of euthanasia. Intestinal blockages appeared to be composed of an inspissated mixture of food material and possibly mucus, with considerable distention of the intestine observed proximally to the blockages. Histologically, some of the *Cftr* ^*Δ9/Δ9*^ mouse intestine sections showed distention with large luminal space, with no other obvious abnormalities (Figures 2C,D). The small intestine sections from *Cftr* ^*Δ9/Δ9*^ mice showed that the crypts of Lieberkühn were dilated (Figure 2E), compared with those from the *Cftr* ^*Δ9/+*^ litter mates (Figure 2F). There were no differences between genders.

Alcian blue stains mucus a bright blue and staining of organ sections revealed that crypts of Lieberkühn in *Cftr* ^*Δ9/Δ9*^ mice contained mucus, however, this did not differ to that of the *Cftr* ^*Δ9/+*^ control mice (Figures 2G,H). *Cftr* ^*Δ9/Δ9*^ did appear to have more goblet cells (Figures 2I,J) than their *Cftr* ^*Δ9/+*^ littermates, however this was not quantified. No macroscopic abnormalities were noted in the spleen, kidneys or liver. Due to the relatively small colony size generated in this study, quantification of goblet cell number or the characterisation of mucus accumulation could not be performed.

### *Cftr* ^*Δ9/Δ9*^ Airway Epithelial Cells

**FIGURE 3:**
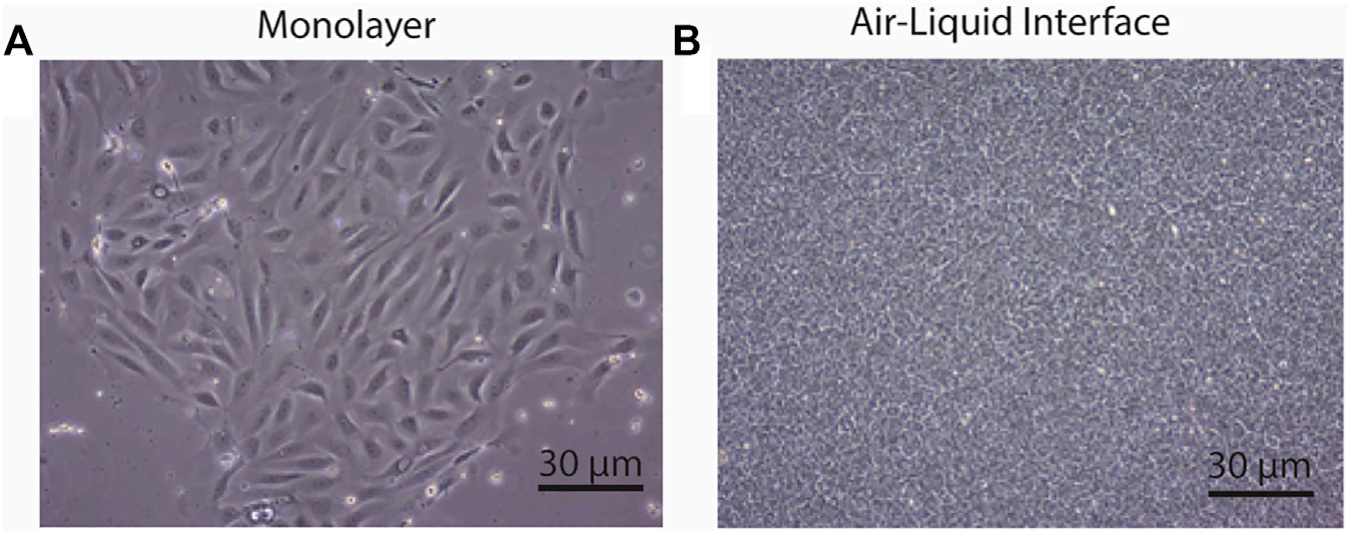
CftrΔ9/Δ9 deleted airway epithelial cell morphology. **(A)**. Airway epithelial cells grown in monolayer had standard cobble stone appearance. Scale bar: 30 micrometres **(B).** Airway epithelial cells grown at the air-liquid interface with the dense packed growth morphology. Scale bar 30 micrometres. *n* = 4.

**FIGURE 4:**
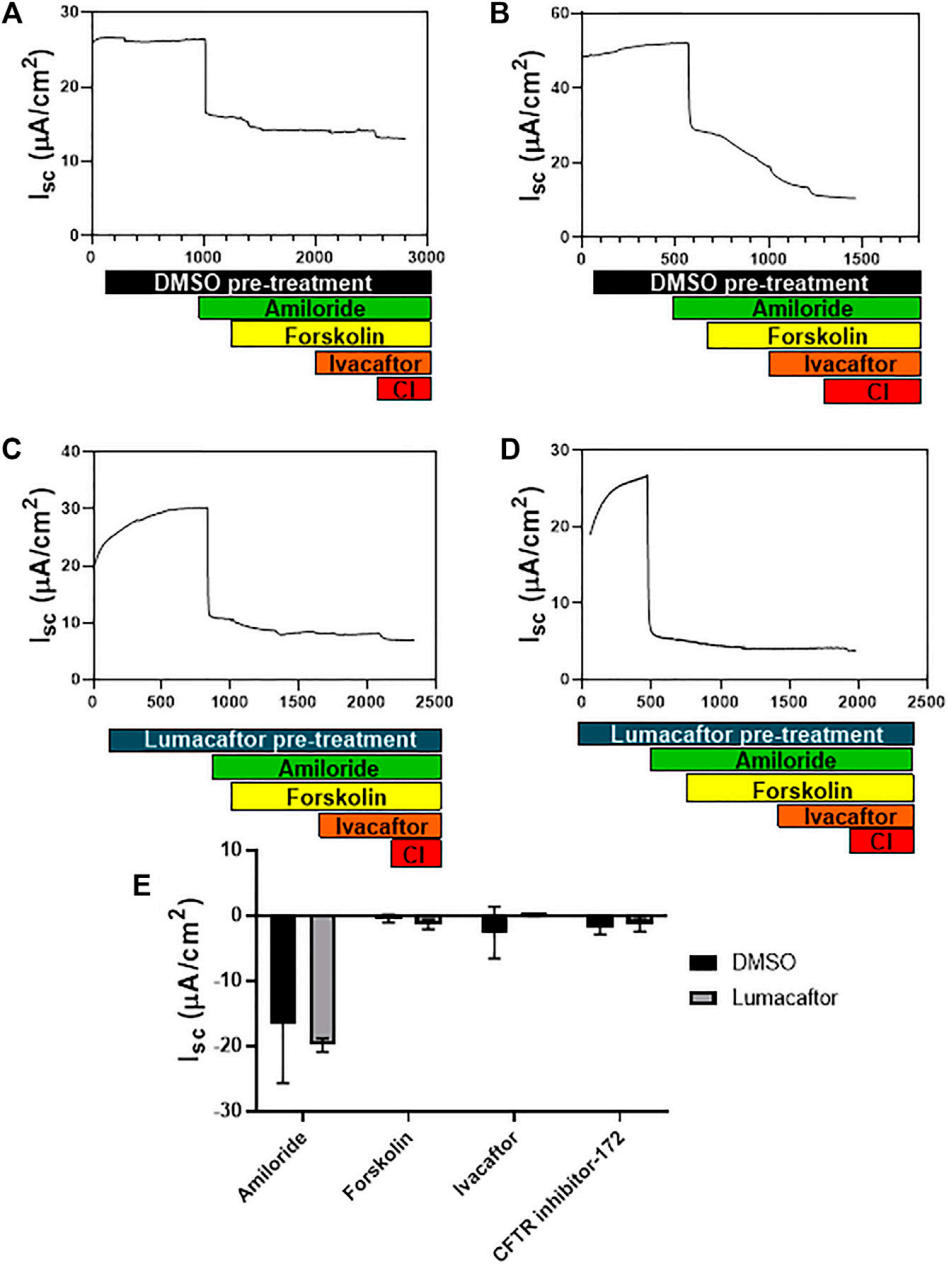
*Cftr* ^*Δ9/Δ9*^ epithelial cell CFTR function. **(A,B)**. Ussing chamber tracing showing change in short circuit current (*Isc*) in response to drug stimulation on *Cftr* ^*Δ9/Δ9*^ ALI cultures pre-treated for 24 h with DMSO. **(C,D)**. Ussing chamber tracing showing change in *Isc* in response to drug stimulation on homozygote *Cftr* exon 9 deleted ALI cultures pre-treated for 24 h with Lumacaftor. **(E)**. Summary of changes in *Isc* in response to amiloride, forskolin and CFTR inhibitor-172 for *Cftr* ^*Δ9/Δ9*^ ALI cultures pre-treated with DMSO (Black bars) or Lumacaftor (Grey bars) (*Isc*, *n* = 2). Pre-treatment with DMSO (black bar) pre-treatment with Lumacaftor (blue bar), Amiloride addition (green bar), Forskolin stimulation (yellow bar), Ivacaftor stimulation (orange bar), CFTR inhibitor-172 (CI-red bar).

Primary airway cultures from *Cftr* ^*Δ9/Δ9*^ deleted mice exhibited typical epithelial cobblestone morphology when grown as a submerged monolayer (Figure 3A, *n* = 4). *Cftr* ^*Δ9/+*^ littermates failed to culture due to contamination, poor cellular adherence to the culture surface therefore this control group is lacking. When grown at the ALI, the *Cftr* ^*Δ9/Δ9*^ pAECs were densely packed with beating cilia (Figure 3B), typical of a pseudostratified mucociliary differentiated culture insert. To assess differentiation, TEER of ALI culture inserts was measured, peaking on day 9 at ∼ 3,100 Ω/cm^2^. As differentiation progressed, the TEER dropped to ∼ 700 Ω/cm^2^ on day 18 and was maintained until Ussing chamber analysis was conducted on day 28 (Martinovich et al., 2017). Primary *Cftr* ^*Δ9/Δ9*^ ALI cultures were pre-treated with either a DMSO control (Figures 4A,B) or Lumacaftor (Figures 4C,D) for 24 h before routine CFTR functional analysis using an Ussing chamber. All *Cftr* ^*Δ9/Δ9*^ ALI cultures demonstrated a drop in short circuit current (*Isc*) in response to amiloride addition, representing the blocking of sodium adsorption (DMSO: -16.5 ± 9.2 μA/cm^2^, Lumacaftor: -19.9 ± 1.061 μA/cm^2^, *n* = 2). In addition, *Cftr* ^*Δ9/Δ9*^ ALI cultures showed a very minimal response to forskolin stimulation (DMSO: -0.5 ± 0.6 μA/cm^2^, Lumacaftor: -1.4 ± 0.8 μA/cm^2^, *n* = 2), negative response to Ivacaftor (DMSO: -2.6 ± 3.9 μA/cm^2^, Lumacaftor: -0.5 ± 0.1 μA/cm^2^, *n* = 2) and minimal response to CFTR inhibitor-172 (DMSO: -1.8 ± 1.1 μA/cm^2^, Lumacaftor: *-*1.3 ± 1.1 μA/cm^2^, *n* = 2) (Figure 4E).

## Discussion

Here, the literature was interrogated for an exon that harboured multiple *CFTR* mutations that presented as a milder form of CF disease (ie CBAVD) indicating that the exon could be partially redundant, with *CFTR* exon 9 being identified. To investigate the *in vitro* and *in vivo* functionality of a CFTR isoform missing the domain encoded by exon 9, a *Cftr* ^*Δ9/Δ9*^ mouse model was generated. Although *Cftr* mouse models do not develop CF lung disease, this model was used to ascertain if the level of disease severity correlated with *Cftr* genotype. It was hypothesised that mice lacking *Cftr* exon 9 alone would present with less severe pathological disease, when compared to other models, including *Cftr* gene KO or mutations resulting in premature termination of translation and nonsense mediated decay (Ratcliff et al., 1993; Colledge et al., 1995; Hasty et al., 1995). The *Cftr* ^*Δ9/Δ9*^ mouse model generated here by homologous recombination presented with intestinal obstructions that were not prevented or improved by a liquid diet. At time of weaning (21 days) *Cftr* ^*Δ9/Δ9*^ mice had a survival rate of 83% that dropped to 38% by day 50, with most mice eventually reaching a humane endpoint as described in the approved ethics protocol due to intestinal obstruction or malnutrition. Finally, airway epithelial cell cultures established from *Cftr* ^*Δ9/Δ9*^ mouse airways were not responsive to the CFTR stimulator drug forskolin. Collectively, data generated in this study suggest that *Cftr* exon 9 is not partially redundant in mice, although airway cells from *Cftr* ^*Δ9/+*^ littermates failed to culture due to contamination, poor adherence to the culture surface therefore this vital control group is lacking.

Survival of the *Cftr* ^*Δ9/Δ9*^ mice generated here was initially high at 83% in the first 21 days, compared to the 21 days survival of published *Cftr* KO mouse models with no residual WT mRNA, such as the *Cftr* ^tm1Unc^ null model with ∼25% survival (Snouwaert et al., 1992), and the *Cftr* ^tm1Cam^ null with only 17% (Ratcliff et al., 1993). *Cftr* mouse models with missense mutations and residual WT *Cftr* mRNA, such as *Cftr* ^tm/Bay^ has 40% and *Cftr* ^tm/Hgu^, has 90% survival at 21 days. This could suggest that the disease severity of the *Cftr* ^*Δ9/Δ9*^ mouse model generated here was marginally less severe with respect to intestinal complications. Homologous recombination mouse models as developed here, overcome any confounding issues by having no residual WT *Cftr* mRNA present that could potentially mitigate the disease presentation. As the *Cftr* exon 9 deletion occurred in the genome and is the only source of the *Cftr* gene expressed in this homozgygous mouse model, there will be no residual full-length *Cftr* that could influence the survival rate. In addition to specific genetic differences, variations in husbandry and housing environment such as the use of Peptamen® and ALPHA-dri® bedding as used here may also influence survival rates between mouse models.

There are many phenotypic differences between different mouse models of CF, due to background strain, environment and the specific *Cftr* mutation. Like many other animal models (e.g., the *mdx* mouse and Duchenne muscular dystrophy), CF mouse models do not accurately reflect the lung disease that occurs in humans, and our model was no different. Histological examination of organs from *Cftr* ^*Δ9/Δ9*^ mice and heterozygous control littermates revealed few differences. There were no notable changes in the lungs, kidneys or liver evident on Hematoxylin and Eosin stained sections, which is comparable with many *Cftr* mouse models (Scholte et al., 2004). The organs, including the small intestines were also investigated for structural differences using Hematoxylin and Eosin staining of sections and examining mucus accumulation *via* Alcian blue staining. Examination of these sections from *Cftr* ^*Δ9/Δ9*^ mice appeared to have an increase in the number of goblet cells with a swollen lumen and dilated Crypts of Lieberkühn, when compared to heterozygous littermates although quantification could not be done. Alcian blue staining showed dilated goblet cells and Crypts of Lieberkühn that were mucus filled. These findings reflect those from other CF mouse models presenting with intestinal complications and no lung disease (Wilke et al., 2011).

Airway epithelial cells were successfully isolated from homozygous *Cftr* ^*Δ9/Δ9*^ mice and differentiated ALI cultures were established. The ALI cultures developed beating cilia, produced mucous and differentiated, as supported by high TEER values. Ussing chamber studies on ALI cultures revealed that these *Cftr* ^*Δ9/Δ9*^ ALI cultures had a good response to amiloride, indicating sodium channels were present and blocked by the addition of this drug. The *Cftr* ^*Δ9/Δ9*^ ALI cultures did not respond to forskolin stimulation, indicating that no CFTR channels were opening in response to the drug. There was minimal response to the CFTR inhibitor-172 as expected, and the small change seen could be due to volume-sensitive outwardly rectifying chloride ion conductance (Melis et al., 2014). These results indicate that the CFTR protein produced from the *Cftr* mRNA lacking exon 9 did not have the functional capacity to respond to forskolin, and therefore was a non-functional CFTR channel. When investigating this finding it was noted that electrophysical responses differ depending on the mouse strain, since it is known that other factors such as genetic background can influence this response (Davidson and Rolfe 2001; Wilke et al., 2011). For example, *Cftr* ^tm1Cam^ C57Bl/6 mice have a forskolin response that is comparable to WT mice, whereas *Cftr* ^tm1Cam^ mice on an FVB background have no forskolin response (Wilke et al., 2011). The difference between our *Cftr* ^*Δ9/Δ9*^ C57Bl/6 cell model lacking forskolin response and the *Cftr* ^tm1Cam^ C57Bl/6 model that had a forskolin response could be due to differences in the functional measurements. Here, pAEC ALI cultures were established *in vitro* from bronchial epithelium to measure the forskolin response, whereas Wilke et al (2011) utilised excised nasal epithelial sheets for Ussing chamber analysis (Wilke et al., 2011). This confounding issue reflects some of the problems encountered when utilising animal models to represent human disease and different methods to capture functional data.

Given the intermediate survival rate seen with diet supplemented (from 21 days) *Cftr* ^*Δ9/Δ9*^ mice, *Cftr* exon 9 could have some minor level of redundancy, at least in this mouse model. Duchenne muscular dystrophy is an example of genotype/phenotype correlations; and the *DMD* gene contains a number of partially dispensable exons, as we and others have observed some functional protein is preferable to no functional protein (Mann et al., 2001). This redundancy may be of therapeutic potential and future work should utilise human tissue to investigate this further. This mouse model like others could be used to bridge the gap between cell culture-based studies and human trials for evaluation of novel drug efficacy and dosing. This *Cftr* ^*Δ9/Δ9*^ mouse model demonstrates that exon skipping could be a feasible strategy for selected *CFTR* mutations to reduce a severe CF phenotype, although further investigation in human-derived tissue models is required as has been done for CFTR exon 23 skipping for mutations p.Trp1282X (Kim et al., 2022) (Oren et al., 2021; Michaels et al., 2022).

## Data Availability

The raw data supporting the conclusions of this article will be made available by the authors, without undue reservation.

## References

[B1] Clarke L. L. Grubb B. R. Yankaskas J. R. Cotton C. U. McKenzie A. Boucher R. C. (1994). Relationship of a Non-cystic Fibrosis Transmembrane Conductance Regulator-Mediated Chloride Conductance to Organ-Level Disease in Cftr(-/-) Mice. Proc. Natl. Acad. Sci. U S A. 91 (2), 479–483. 10.1073/pnas.91.2.479 7507247 PMC42972

[B2] Colledge W. H. Abella B. S. Southern K. W. Ratcliff R. Jiang C. Cheng S. H. (1995). Generation and Characterization of a delta F508 Cystic Fibrosis Mouse Model. Nat. Genet. 10 (4), 445–452. 10.1038/ng0895-445 7545494

[B3] Cottart C. H. Bonvin E. Rey C. Wendum D. Bernaudin J. F. Dumont S. (2007). Impact of Nutrition on Phenotype in CFTR-Deficient Mice. Pediatr. Res. 62 (5), 528–532. 10.1203/PDR.0b013e318155a61d 17805210

[B4] Davidson D. J. Rolfe M. (2001). Mouse Models of Cystic Fibrosis. Trends Genet. 17 (10), S29–S37. 10.1016/s0168-9525(01)02452-0 11585674

[B5] Gaikwad A. Khan S. Kadam S. Kadam K. Dighe V. Shah R. (2018). The CFTR Gene Mild Variants Poly-T, TG Repeats and M470V Detection in Indian Men with Congenital Bilateral Absence of Vas Deferens. Andrologia 50 (2), e12858. 10.1111/and.12858 28776713

[B6] Gosselin D. Stevenson M. M. Cowley E. A. Griesenbach U. Eidelman D. H. Boulé M. (1998). Impaired Ability of Cftr Knockout Mice to Control Lung Infection with *Pseudomonas aeruginosa* . Am. J. Respir. Crit. Care Med. 157 (4 Pt 1), 1253–1262. 10.1164/ajrccm.157.4.9702081 9563748

[B7] Hasty P. O'Neal W. K. Liu K. Q. Morris A. P. Bebok Z. Shumyatsky G. B. (1995). Severe Phenotype in Mice with Termination Mutation in Exon 2 of Cystic Fibrosis Gene. Somat Cel Mol Genet 21 (3), 177–187. 10.1007/BF02254769 7482032

[B8] Henrie A. Hemphill S. E. Ruiz-Schultz N. Cushman B. DiStefano M. T. Azzariti D. (2018). ClinVar Miner: Demonstrating Utility of a Web-Based Tool for Viewing and Filtering ClinVar Data. Hum. Mutat. 39 (8), 1051–1060. 10.1002/humu.23555 29790234 PMC6043391

[B9] Kim Y. J. Sivetz N. Layne J. Voss D. M. Yang L. Zhang Q. (2022). Exon-skipping Antisense Oligonucleotides for Cystic Fibrosis Therapy. Proc. Natl. Acad. Sci. 119 (3), e2114858118. 10.1073/pnas.2114858118 35017301 PMC8784140

[B10] Koentgen F. Lin J. Katidou M. Chang I. Khan M. Watts J. (2016). Exclusive Transmission of the Embryonic Stem Cell-Derived Genome through the Mouse Germline. genesis 54 (6), 326–333. 10.1002/dvg.22938 27012318 PMC5084746

[B11] Lam H. C. Choi A. M. Ryter S. W. (2011). Isolation of Mouse Respiratory Epithelial Cells and Exposure to Experimental Cigarette Smoke at Air Liquid Interface. J. Vis. Exp. 48, 2513. 10.3791/2513 PMC3197407 21372793

[B12] Lavelle G. M. White M. M. Browne N. McElvaney N. G. Reeves E. P. (2016). Animal Models of Cystic Fibrosis Pathology: Phenotypic Parallels and Divergences. Biomed. Res. Int. 2016, 5258727. 10.1155/2016/5258727 27340661 PMC4908263

[B13] Li H. Wen Q. Li H. Zhao L. Zhang X. Wang J. (2012). Mutations in the Cystic Fibrosis Transmembrane Conductance Regulator (CFTR) in Chinese Patients with Congenital Bilateral Absence of Vas Deferens. J. Cyst Fibros 11 (4), 316–323. 10.1016/j.jcf.2012.01.005 22483971

[B14] Mann C. J. Honeyman K. Cheng A. J. Ly T. Lloyd F. Fletcher S. (2001). Antisense-induced Exon Skipping and Synthesis of Dystrophin in the Mdx Mouse. Proc. Natl. Acad. Sci. U S A. 98 (1), 42–47. 10.1073/pnas.011408598 11120883 PMC14541

[B15] Martinovich K. M. Iosifidis T. Buckley A. G. Looi K. Ling K. M. Sutanto E. N. (2017). Conditionally Reprogrammed Primary Airway Epithelial Cells Maintain Morphology, Lineage and Disease Specific Functional Characteristics. Sci. Rep. 7 (1), 17971. 10.1038/s41598-017-17952-4 29269735 PMC5740081

[B16] Melis N. Tauc M. Cougnon M. Bendahhou S. Giuliano S. Rubera I. (2014). Revisiting CFTR Inhibition: a Comparative Study of CFTRinh -172 and GlyH-101 Inhibitors. Br. J. Pharmacol. 171 (15), 3716–3727. 10.1111/bph.12726 24758416 PMC4128068

[B17] Michaels W. E. Pena-Rasgado C. Kotaria R. Bridges R. J. Hastings M. L. (2022). Open reading Frame Correction Using Splice-Switching Antisense Oligonucleotides for the Treatment of Cystic Fibrosis. Proc. Natl. Acad. Sci. 119 (3), e2114886119. 10.1073/pnas.2114886119 35017302 PMC8784102

[B18] Molinski S. V. Shahani V. M. Subramanian A. S. MacKinnon S. S. Woollard G. Laforet M. (2018). Comprehensive Mapping of Cystic Fibrosis Mutations to CFTR Protein Identifies Mutation Clusters and Molecular Docking Predicts Corrector Binding Site. Proteins 86 (8), 833–843. 10.1002/prot.25496 29569753

[B19] O′Neal W. K. Hasty P. McCray P. B. Jr Casey B. Rivera-Pérez J. Welsh M. J. (1993). A Severe Phenotype in Mice with a Duplication of Exon 3 in the Cystic Fibrosis Locus. Hum. Mol. Genet. 2 (10), 1561–1569. 10.1093/hmg/2.10.1561 7505691

[B20] Oren Y. S. Avizur-Barchad O. Ozeri-Galai E. Elgrabli R. Schirelman M. R. Blinder T. (2021). Antisense Oligonucleotide Splicing Modulation as a Novel Cystic Fibrosis Therapeutic Approach for the W1282X Nonsense Mutation. J. Cystic Fibrosis S1569-1993, 02172. 10.1016/j.jcf.2021.12.012 34972649

[B21] Radpour R. Gourabi H. Gilani M. A. Dizaj A. V. (2007). Molecular Study of (TG)m(T)n Polymorphisms in Iranian Males with Congenital Bilateral Absence of the Vas Deferens. J. Androl. 28 (4), 541–547. 10.2164/jandrol.106.002337 17314234

[B22] Ratcliff R. Evans M. J. Cuthbert A. W. MacVinish L. J. Foster D. Anderson J. R. (1993). Production of a Severe Cystic Fibrosis Mutation in Mice by Gene Targeting. Nat. Genet. 4 (1), 35–41. 10.1038/ng0593-35 7685652

[B23] Rozmahel R. Wilschanski M. Matin A. Plyte S. Oliver M. Auerbach W. (1996). Modulation of Disease Severity in Cystic Fibrosis Transmembrane Conductance Regulator Deficient Mice by a Secondary Genetic Factor. Nat. Genet. 12 (3), 280–287. 10.1038/ng0396-280 8589719

[B24] Scholte B. J. Davidson D. J. Wilke M. De Jonge H. R. (2004). Animal Models of Cystic Fibrosis. J. Cyst Fibros 3 Suppl 2 (Suppl. 2), 183–190. 10.1016/j.jcf.2004.05.039 15463956

[B25] Shteinberg M. Downey D. G. Beattie D. McCaughan J. Reid A. Stein N. (2017). Lung Function and Disease Severity in Cystic Fibrosis Patients Heterozygous for p.Arg117His. ERJ Open Res. 3 (1), 00056–02016. 10.1183/23120541.00056-2016 PMC5566269 28845426

[B26] Snouwaert J. N. Brigman K. K. Latour A. M. Malouf N. N. Boucher R. C. Smithies O. (1992). An Animal Model for Cystic Fibrosis Made by Gene Targeting. Science 257 (5073), 1083–1088. 10.1126/science.257.5073.1083 1380723

[B27] Thomas E. J. Gabriel S. E. Makhlina M. Hardy S. P. Lethem M. I. (2000). Expression of Nucleotide-Regulated Cl(-) Currents in CF and normal Mouse Tracheal Epithelial Cell Lines. Am. J. Physiol. Cel Physiol 279 (5), C1578–C1586. 10.1152/ajpcell.2000.279.5.C1578 11029305

[B28] Toronto C. F. C. A. T. H. F. S. C. I. (2011). Cystic Fibrosis Mutation Database (CFTR1). Retrieved from http://www.genet.sickkids.on.ca/ .(Accessed 22/08, 2020)

[B29] Wilke M. Buijs-Offerman R. M. Aarbiou J. Colledge W. H. Sheppard D. N. Touqui L. (2011). Mouse Models of Cystic Fibrosis: Phenotypic Analysis and Research Applications. J. Cyst Fibros 10 Suppl 2 (Suppl. 2), S152–S171. 10.1016/S1569-1993(11)60020-9 21658634

[B30] Zeiher B. G. Eichwald E. Zabner J. Smith J. J. Puga A. P. McCray P. B. Jr. (1995). A Mouse Model for the delta F508 Allele of Cystic Fibrosis. J. Clin. Invest. 96 (4), 2051–2064. 10.1172/JCI118253 7560099 PMC185844

[B31] Zhou Z. Duerr J. Johannesson B. Schubert S. C. Treis D. Harm M. (2011). The ENaC-Overexpressing Mouse as a Model of Cystic Fibrosis Lung Disease. J. Cyst Fibros 10 Suppl 2 (Suppl. 2), S172–S182. 10.1016/S1569-1993(11)60021-0 21658636

